# Effect of Build Orientation on the Electrical, Thermal, and Mechanical Properties of PA12 Fabricated by Selective Laser Sintering

**DOI:** 10.3390/ma19204329

**Published:** 2026-10-15

**Authors:** Le Huang, Sirui Tu, Yang Lyu, Yanxin Tu, Han Li

**Affiliations:** 1 State Key Laboratory of Disaster Prevention and Reduction for Power Grid, Disaster Prevention and Reduction Center of State Grid Hunan Electric Power Co., Ltd., Changsha 410100, China; huangle_@whu.edu.cn (L.H.);; 2 School of Electrical Engineering and Automation, Wuhan University, Wuhan 430072, China; tusirui@whu.edu.cn (S.T.);

**Keywords:** selective laser sintering, build orientation, anisotropy, electrical, thermal, and mechanical properties, high-voltage insulation

## Abstract

Selective laser sintering (SLS) provides a promising route for the rapid fabrication of polymeric insulating components with complex geometries. However, its layer-by-layer manufacturing process inherently introduces structural anisotropy, which may significantly affect the electrical, thermal, and mechanical properties of the printed materials. In this study, polyamide 12 (PA12) specimens fabricated by SLS at different printing orientations were systematically investigated for dielectric properties, volume resistivity, DC and AC breakdown strength, tensile properties, and thermal conductivity. Scanning electron microscopy and X-ray microscopy were further employed to characterize the microstructure and clarify the origin of the anisotropic behavior. The results show that SLS-printed PA12 exhibits pronounced orientation-dependent properties. As the printing angle increases from 0° to 90°, the volume resistivity and DC breakdown strength generally decrease. In contrast, the relative permittivity generally increases with printing angle, whereas the AC breakdown strength and dielectric loss exhibit more complex non-monotonic variations. The tensile strength and elongation at break both gradually decrease with increasing printing angle. The thermal conductivity varies only slightly across different orientations but increases overall with printing angle. Microstructural observations reveal numerous interlayer pores, incompletely sintered regions, and heterogeneous interfaces with a distinct layered distribution. These heterogeneous interlayer structures affect the transport of charge, heat, and load, thereby contributing to the anisotropic electrical, thermal, and mechanical properties of SLS-printed PA12. These findings provide a basis for optimizing the printing orientation and structural design of SLS-printed PA12 for high-voltage insulation applications.

## 1. Introduction

In the field of high-voltage insulation, improving the electrical withstand capability of insulating equipment generally requires comprehensive optimization of material systems, structural designs, and functional performance. However, the increasing demand for complex structures and enhanced performance also poses significant challenges to conventional manufacturing technologies [1]. Meanwhile, high-voltage live-line operation tools often require customized structures due to their specific service scenarios while maintaining excellent mechanical, electrical, and thermal properties [2]. Recently, three-dimensional (3D) printing technology has attracted increasing attention in the field of electrical insulation due to its high design flexibility, rapid prototyping capability, and advantages in customized manufacturing [3,4,5]. Unlike conventional manufacturing processes, 3D printing enables precise structural regulation and fabrication of complex geometries and functionally graded materials, providing new opportunities for the development of advanced insulating materials and equipment.

Currently, several 3D printing technologies have been explored for fabricating insulating materials, including stereolithography (SLA), selective laser sintering (SLS), and fused deposition modeling (FDM) [6,7]. Among them, SLS is one of the most widely investigated additive manufacturing technologies. In SLS, polymer powder particles are selectively melted and fused by a high-energy laser beam, forming a 3D structure layer by layer [8]. The unmelted powder surrounding the fabricated part can serve as a natural support material, eliminating the requirement for additional support structures. Compared with SLA and FDM, SLS provides greater design freedom, improved dimensional accuracy, and superior mechanical performance [9]. Moreover, it is better suited to fabricating large, complex components, making it a promising technology for high-voltage insulation applications.

Nevertheless, several challenges remain for the practical application of SLS technology. Since SLS employs a layer-by-layer manufacturing strategy, adjacent layers are bonded primarily through interfacial fusion of molten powder particles [10]. Consequently, SLS-printed materials usually exhibit lower structural density than injection-molded materials and may contain internal defects such as pores. Furthermore, the dependence of material properties on laser scanning paths and interlayer bonding mechanisms can introduce anisotropy, leading to variations in performance along different printing directions [11,12]. Therefore, understanding how build orientation affects material properties is essential for the application of SLS-printed insulating materials.

Several studies have investigated the anisotropic behavior of SLS-printed polymers. Psarros et al. evaluated the tensile and compressive properties of SLS-fabricated polyamide 12 (PA12) specimens across different thicknesses, build orientations, and loading rates. Their results demonstrated that build orientation had the most significant influence on mechanical properties, and tensile properties were more sensitive than compressive properties [13]. Slager et al. demonstrated that the degree of powder melting, interlayer bonding quality, and structural defects strongly affected the mechanical anisotropy of SLS-printed materials [14]. Wang et al. analyzed the effects of laser energy density and printing angle on the electrical properties of SLS-fabricated PA12 and found that these parameters could significantly modify the material’s electrical characteristics [15]. These studies indicate that SLS processing parameters can influence not only mechanical properties but also electrical properties. However, existing studies on the anisotropy of SLS-printed materials still have several limitations. First, most investigations focus on only a few typical build orientations (e.g., 0°, 45°, and 90°), while systematic analysis of the continuous evolution of orientation-dependent properties remains insufficient. Second, previous studies generally concentrate on a single performance aspect, such as mechanical or electrical properties, without comprehensively evaluating the multiple properties relevant to high-voltage insulation applications. Moreover, the microstructural features responsible for the observed macroscopic property variations have received limited attention, hindering the establishment of clear microstructure–property relationships and the development of rational strategies for build orientation selection and performance optimization. For high-voltage insulation equipment and live-line operation tools, however, materials are simultaneously subjected to electrical stress, thermal effects, and mechanical loading during service. Therefore, evaluating only a single property cannot fully capture their practical performance. Furthermore, defects introduced during the SLS process, particularly interlayer pores and incompletely sintered regions, may interact with the build orientation and consequently contribute to the anisotropic properties of the printed parts. A comprehensive investigation of the electro-thermal–mechanical properties induced by SLS printing processes is therefore necessary.

In this work, the influence of SLS build orientation on the electro-thermal–mechanical properties of printed materials was systematically investigated. Tensile strength, thermal conductivity, dielectric constant, breakdown strength, and volume resistivity were measured to evaluate the orientation-dependent performance of SLS-printed specimens. Furthermore, microscopic characterization and qualitative mechanism analysis were combined to reveal the underlying mechanisms responsible for the observed property variations. The remainder of this paper is organized as follows: Section 2 introduces the principle of SLS printing and experimental procedures. Section 3 presents the experimental results. Section 4 discusses the mechanisms governing the observed phenomena. Finally, conclusions are summarized in Section 5.

## 2. Experimental Methodology

### 2.1. Materials and Specimen Preparation

PA12 exhibits excellent mechanical properties and electrical insulation performance and is one of the most widely used polymer materials in SLS. Owing to its relatively low cost and favorable processability, PA12 also shows considerable potential for applications in high-voltage insulation engineering [16]. Therefore, PA12 was selected as the material investigated in this study to systematically evaluate the effects of build orientation on its electrical, thermal, and mechanical properties.

**Figure 1:**
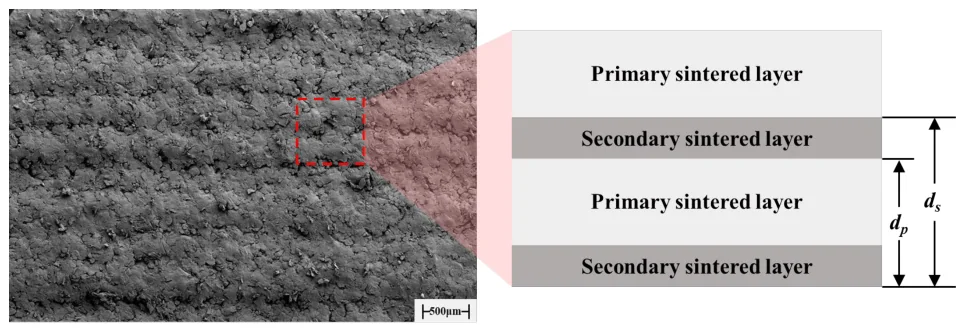
Schematic illustration of the structure of SLS-printed PA12 materials.

During the SLS process, a laser beam selectively scans and sinters the powder bed to form each layer. When a subsequent layer is processed, the laser not only melts and consolidates the newly deposited powder but also partially remelts the upper region of the previously sintered layer, thereby promoting interlayer bonding [17,18]. Consequently, the fabricated specimen inherently exhibits a layered structure. As illustrated in Figure 1, the region of the previously formed layer that undergoes repeated thermal processing is referred to herein as the secondary sintering layer. Here, *d_s_* denotes the effective laser sintering depth, while *d_p_* represents the layer thickness. To ensure sufficient interlayer bonding during fabrication, the layer thickness should generally be smaller than the effective laser sintering depth, i.e., *d_p_* < *d_s_* [19,20]. This layer-by-layer thermal history and the resulting interlayer structure distinguish SLS-fabricated parts from conventionally injection-molded components. The interfaces between adjacent layers, together with repeated melting and solidification, may lead to orientation-dependent microstructures and consequently anisotropic material properties [21,22,23]. Therefore, investigating the electrical, thermal, and mechanical properties of SLS-fabricated PA12 along different build orientations is essential for evaluating its suitability for high-voltage insulation applications.

Commercial PA12 powder (FS3300PA, Farsoon Technologies, Changsha, China), consisting of virgin and recycled powder blended at a mass ratio of 2:8, was used in this study. According to the manufacturer’s specifications, the main properties of PA12 are summarized in Table 1. The heat deflection temperature (HDT) corresponds to the temperature measured under a flexural stress of 1.8 MPa in accordance with ISO 75-1:2020 [24].

A Flight series SLS 3D printing system (Farsoon Technologies, China) was used to fabricate the specimens, and the corresponding printing parameters are listed in Table 2.

To reduce the potential influence of temperature variations along the build direction, specimens representing all seven build orientations were included within each build-height level, and the replicate sets were arranged at subsequent levels. The powder-bed preheating temperature and chamber temperature were maintained at the prescribed settings throughout the build. However, because the in-plane specimen positions were not formally randomized, the possible influence of local thermal gradients across the powder bed cannot be completely excluded.

**Figure 2:**
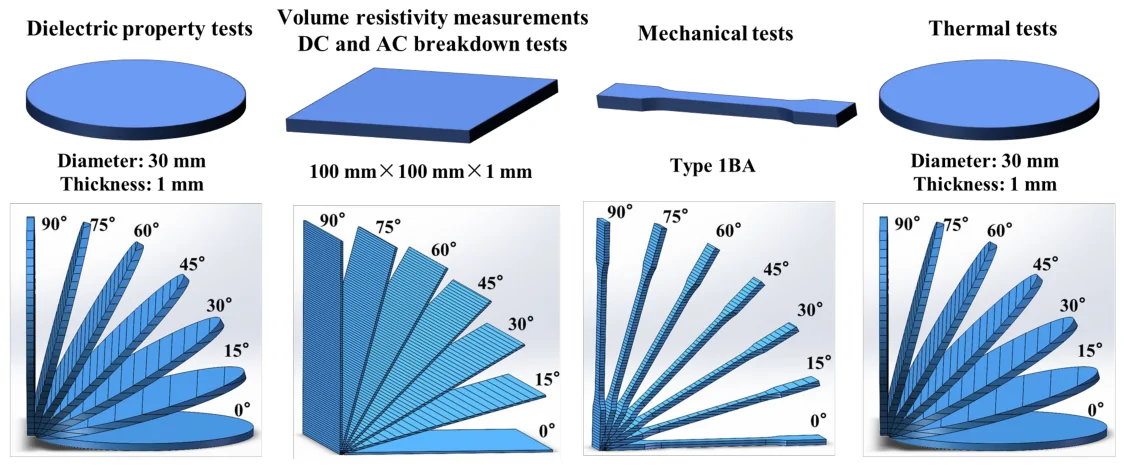
Schematic illustration of the printed specimens and build orientations.

To characterize the electrical, thermal, and mechanical properties of the SLS-fabricated PA12, specimens with different geometries were designed and fabricated, as illustrated in Figure 2. For the electrical tests, circular specimens with a diameter of 30 mm and a thickness of 1 mm were fabricated for dielectric property measurements. Square specimens measuring 100 mm × 100 mm × 1 mm were prepared for volume resistivity measurements and also used for DC and AC breakdown tests. For the mechanical tests, Type 1BA tensile specimens were prepared in accordance with ISO 527-2:2025 [25] to determine the tensile strength and elongation at break. For the thermal tests, the specimens were identical to those used for the dielectric property measurements, with a diameter of 30 mm and a thickness of 1 mm. Thermal conductivity was measured using the transient line-source method.

To investigate the influence of build orientation, the horizontal plane was defined as the reference plane. A build orientation of 0° corresponded to the specimen orientation parallel to the horizontal plane, whereas 90° corresponded to the orientation perpendicular to the horizontal plane and parallel to the build direction. Seven build orientations, ranging from 0° to 90° at intervals of 15° (0°, 15°, 30°, 45°, 60°, 75°, and 90°), were investigated, as shown in Figure 2. The electrical, thermal, and mechanical tests described above were subsequently conducted on specimens fabricated at these orientations.

### 2.2. Experimental Methods

As described above, the electrical, mechanical, and thermal properties of the PA12 specimens fabricated at different build orientations were systematically characterized in accordance with the corresponding standards. The measurements included DC and power-frequency AC breakdown strength, relative permittivity, volume resistivity, tensile properties, and thermal conductivity. The detailed experimental procedures are described below.

#### 2.2.1. Relative Permittivity Measurement

The frequency-dependent relative permittivity was measured using a Concept 80 broadband dielectric spectrometer (Novocontrol Technologies GmbH & Co. KG, Montabaur, Germany) over a frequency range from 0.1 Hz to 1 MHz. All measurements were conducted at an ambient temperature of 25 °C. The specimen was placed between two opposing cylindrical electrodes, each 20 mm in diameter, in a parallel-plate configuration, and the applied voltage was 1 kV. Before each measurement, the electrode surfaces were carefully cleaned to ensure good electrical contact between the specimen and the electrodes. Three specimens were tested for each build orientation, and the curve closest to the mean of the three measurements was selected as the representative curve for analysis.

#### 2.2.2. Volume Resistivity Measurement

According to IEC 62631-3-1:2023 [26], the volume resistivity was measured using a three-electrode configuration with a ZC36 high-resistance meter (Shanghai No.6 Electrical Meter Factory Co., Ltd., Shanghai, China). A DC voltage of 1 kV was applied to the specimen for 600 s. The 600 s electrification time was selected in accordance with the standard and based on the properties of the material. The leakage current was recorded during the measurement, and its average value at the specified measurement duration was used to calculate the volume resistivity of the specimen. Three specimens were tested for each build orientation, and the mean of the three measurements was reported as the final value. The volume resistivity measurements were performed at an ambient temperature of 24 °C and a relative humidity of 57%.

#### 2.2.3. DC and AC Breakdown Tests

The DC breakdown strength was measured using a negative-polarity DC high-voltage generator (Shanghai No.6 Electrical Meter Factory Co., Ltd., Shanghai, China), while the power-frequency AC breakdown test was conducted using a 250 kV/30 kVA partial-discharge-free high-voltage test system. A pair of cylindrical electrodes, each with a diameter of 25 mm and rounded edges with a radius of 2 mm, were used for both tests. The electrodes and specimens were fully immersed in methyl silicone oil during testing to suppress surface discharge and flashover. A continuous-voltage-rise method was used for both the DC and AC breakdown tests, with the voltage increased at a constant rate of 0.5 kV/s until breakdown occurred. For each build orientation, one specimen was used for the DC breakdown test, and another specimen was used for the AC breakdown test. Ten breakdown tests were performed at different locations on each specimen, and the resulting breakdown strength data were analyzed using a two-parameter Weibull distribution.

#### 2.2.4. Tensile Test

The tensile properties were characterized using an MTS 100 kN computer-controlled universal testing machine (MTS Systems Corporation, Eden Prairie, MN, USA). Uniaxial tensile tests were performed at room temperature in accordance with the corresponding standard. The crosshead speed was set to 5 mm/min, and loading was continued until fracture of the specimen. Five specimens were tested for each printing orientation, and the tensile strength and elongation at break were determined from the measured stress–strain curves.

#### 2.2.5. Thermal Tests

The thermal conductivity of the specimens was measured in accordance with ASTM D5930 [27] using a TC3000E thermal conductivity analyzer (XIATECH, Xi’an, China) based on the transient line-source method. During the test, a 9P3000E sensor was placed between two specimens with identical dimensions and flat surfaces to ensure sufficient contact and minimize the influence of interfacial thermal resistance. The voltage applied to the sensor was 1 V, and the sampling duration was 3 s. Three specimens were tested for each build orientation, and their mean value was reported as the thermal conductivity for that orientation.

## 3. Results and Discussion

### 3.1. Dielectric Properties

**Figure 3:**
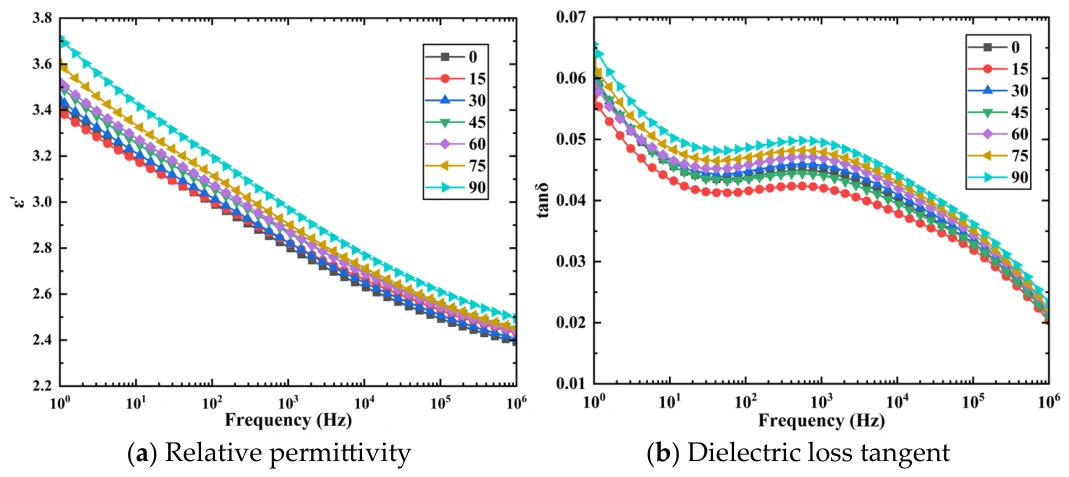
Dielectric properties of specimens with different build orientations: (**a**) relative permittivity and (**b**) dielectric loss tangent. Representative spectra from three repeated measurements (n = 3) are shown for each build orientation.

The experimentally measured relative permittivity (ε′) and dielectric loss tangent (tan δ) are presented in Figure 3. Three specimens were measured for each build orientation, and the repeated measurements exhibited consistent frequency-dependent trends. Representative spectra are presented in Figure 3 for clarity. PA12 specimens fabricated at different build orientations exhibit clear frequency-dependent dielectric behavior. The relative permittivity decreases continuously with increasing frequency, whereas the dielectric loss tangent decreases rapidly in the low-frequency region, exhibits a weak relaxation feature in the intermediate-frequency range, and then decreases further at higher frequencies.

The frequency dependence of the relative permittivity reflects contributions from several polarization and charge-transport processes. PA12 contains polar amide groups (–CONH–), which contribute to dipolar polarization. At low frequencies, however, the relatively high measured ε′ should not be attributed solely to intrinsic dipolar polarization. Slow interfacial polarization, space-charge accumulation, and electrode polarization can also contribute to the apparent permittivity because sufficient time is available for charge accumulation under the alternating electric field. As the frequency increases, these slow polarization and charge-accumulation processes are progressively suppressed, while slower dipolar responses become increasingly unable to follow the rapidly varying electric field, resulting in the continuous decrease in ε′. Therefore, the decrease in ε′ from approximately 3.8 at low frequency to approximately 2.4 at high frequency represents the combined effects of dielectric dispersion and low-frequency charge/electrode-polarization contributions, which cannot be quantitatively separated using the present spectra alone.

A similar frequency-dependent behavior is observed for the dielectric loss tangent. In the low-frequency region, space-charge effects, interfacial polarization, electrode polarization, and slow dipolar responses contribute to dielectric loss. With increasing frequency, these slow processes become progressively less responsive to the alternating electric field, resulting in the initial decrease in tanδ. A weak and broad feature is observed at approximately 10^2^~10^3^ Hz, which may arise from overlapping contributions from dipolar relaxation, molecular chain motions in the amorphous regions, and interfacial polarization associated with the heterogeneous microstructure of SLS-fabricated PA12. Because this feature does not constitute a well-resolved relaxation peak, the individual relaxation processes cannot be reliably separated from the present spectra, and quantitative relaxation parameters were therefore not extracted.

The build orientation also affects the dielectric response of SLS-fabricated PA12. Overall, ε′ tends to increase with increasing printing angle, with the 90° specimen exhibiting the highest values and the 0° specimen generally exhibiting the lowest values. This anisotropic behavior may be closely associated with the secondary sintered regions and interlayer pores formed during the SLS process. For the 0° specimen, where the electric field is approximately perpendicular to the printed layers, the low-permittivity interlayer pores and secondary sintered regions are distributed approximately in series along the electric field direction, thereby reducing the material’s effective permittivity. As the build orientation changes from 0° to 90°, the printed layers become progressively more parallel to the electric field, which may reduce the influence of interlayer interfaces on the effective dielectric response. In comparison, although tan δ is also affected by build orientation, its variation with printing angle is not strictly monotonic. Porosity, interlayer fusion, and polarization processes may all contribute to this variation, and their individual contributions cannot be separated using the present dielectric spectra alone.

### 3.2. Volume Resistivity Properties

**Figure 4:**
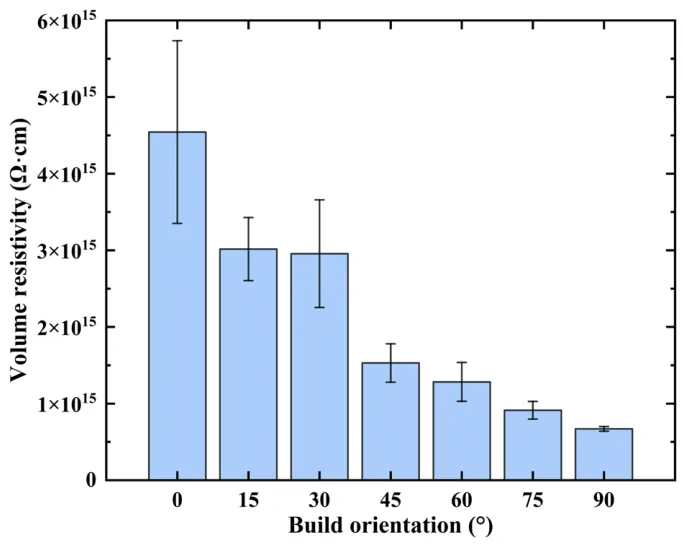
Volume resistivity results of specimens with different build orientations.

Figure 4 shows the variation in the volume resistivity of SLS-printed PA12 specimens at different build orientations. The horizontally printed (0°) specimens exhibit the highest volume resistivity, considerably higher than that of the inclined and vertically printed specimens. Notably, the dependence of volume resistivity on printing angle is nonlinear. In particular, the volume resistivity of the 0° specimen is approximately 6.8 times that of the 90° specimen. One-way ANOVA of the log_10_-transformed data confirmed an overall effect of build orientation. However, adjacent orientations did not all differ significantly; for example, the difference between 15° and 30° was not significant (Tukey’s test, adjusted *p* > 0.999). These results indicate an orientation-dependent trend in volume resistivity without implying a significant change at every 15° increment.

This electrical anisotropy is mainly attributed to the heterogeneous layered structure generated during the SLS process. For the 0° specimen, the applied electric field is approximately perpendicular to the build layers. Consequently, charge carriers migrating along the direction of the electric field must successively cross the primary and secondary sintered layers and multiple interlayer interfaces. Interlayer pores, incompletely fused regions, and interfacial trap states can collectively introduce potential barriers to charge transport, while carrier trapping further inhibits charge migration, resulting in a relatively high volume resistivity. As the printing angle increases, an electric field component parallel to the build layers gradually develops, allowing charge carriers to migrate preferentially within the sintered layers or along the interlayer regions without repeatedly crossing multiple interfaces. This reduces the barrier effect of the interlayer structure and facilitates charge transport. Therefore, even a moderate change in printing angle may substantially alter the effective charge transport pathways, leading to a rapid increase in electrical conductivity and the observed nonlinear dependence of volume resistivity on build orientation.

### 3.3. DC and AC Breakdown Properties

A two-parameter Weibull distribution was used to analyze the DC and AC breakdown characteristics of specimens printed at different build orientations. The cumulative probability of breakdown at an electric field E is expressed as

(1) $$F\left(E;\alpha ,\beta \right)=1-\exp\left(-{\left(\frac{E}{\alpha }\right)}^{\beta }\right)$$

where *E* is the breakdown strength, *α* is the scale parameter corresponding to the characteristic breakdown strength at a cumulative breakdown probability of 63.2%, and *β* is the shape parameter describing the dispersion of the breakdown data.

#### 3.3.1. DC Breakdown Properties

**Figure 5:**
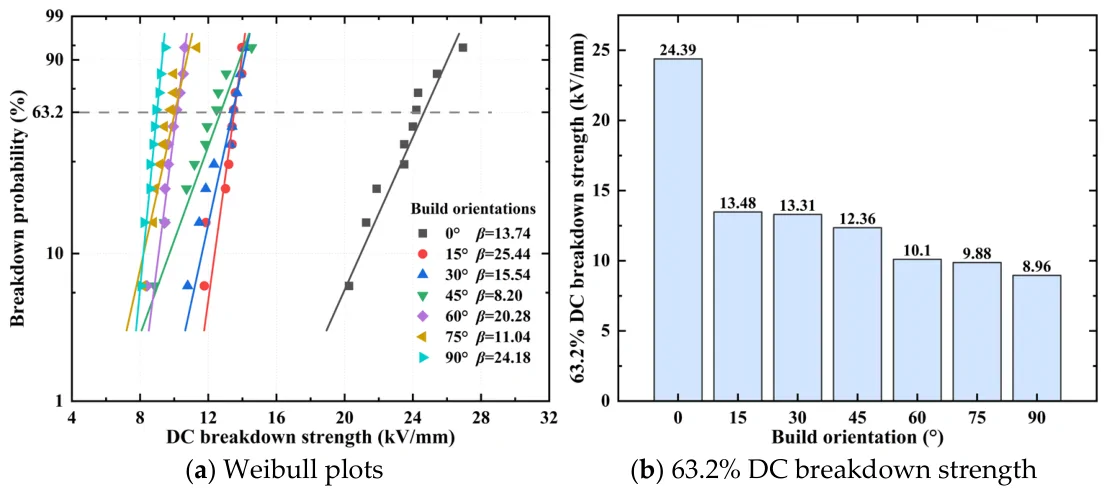
DC breakdown results of specimens with different build orientations.

The DC breakdown results are shown in Figure 5, with the Weibull shape parameter (β) for each build orientation provided in the figure legend. The goodness of fit was assessed using the modified Kolmogorov–Smirnov test. The *p*-values for all build orientations exceeded 0.1; therefore, the Weibull distribution hypothesis was not rejected at the 0.05 significance level. The Weibull scale (α) and shape parameters, together with their 95% confidence intervals, for DC breakdown strength are presented in Table 3. A pronounced anisotropy in DC breakdown strength can be observed with different build orientations. The characteristic DC breakdown strength generally decreases with increasing printing angle, from 24.39 kV/mm at 0° to 8.96 kV/mm at 90°. This trend is highly consistent with that observed for the volume resistivity, suggesting that charge-carrier transport, regulated by the layered structure of the SLS-fabricated material, plays an important role in the DC breakdown process. In addition, the breakdown strength is relatively low at some build orientations. Further improvements in both the printing process and the material are needed before the printed parts can be used in high-voltage applications.

It is noteworthy that while the 0° specimen demonstrates the highest DC breakdown strength, its breakdown strength data show relatively large scatter. This dispersion is likely attributable to the stochastic nature of defect distribution inherent in the SLS process. Specifically, when the breakdown path propagates across multiple interlayer interfaces, the overall breakdown behavior is governed not only by the cumulative interfacial barrier effect but also by the location and severity of local weak interfaces, pores, and insufficiently fused regions. Variations in these randomly distributed defects among individual specimens may therefore lead to substantial differences in local electric-field distribution and breakdown-path development, resulting in greater scatter in the measured breakdown strength.

#### 3.3.2. AC Breakdown Properties

**Figure 6:**
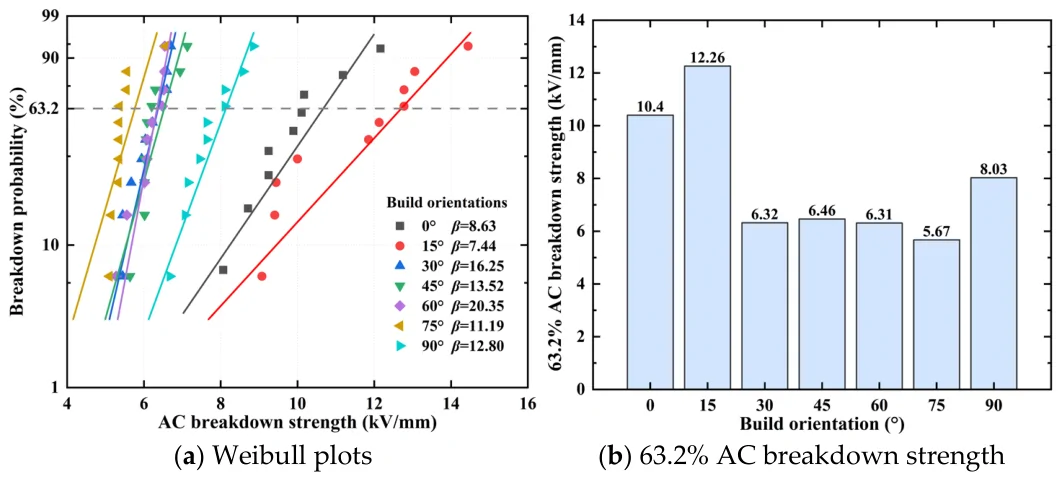
AC breakdown results of specimens with different build orientations.

The AC breakdown results are presented in Figure 6, with the Weibull shape parameters (β) provided in the figure legend. As with the DC breakdown results, the modified Kolmogorov–Smirnov test yielded *p*-values greater than 0.1 for all build orientations. The Weibull parameters and their 95% confidence intervals for AC breakdown strength are presented in Table 4. Similar to the DC case, the AC breakdown performance of SLS-printed PA12 exhibits pronounced orientation dependence; however, its variation with printing angle is non-monotonic. The 0° and 15° specimens exhibit relatively high AC breakdown strengths but comparatively large scatter, whereas the specimens printed at 30–75° show lower breakdown strengths with more concentrated distributions. When the printing angle further increases to 90°, the AC breakdown strength increases slightly. As with DC breakdown, the breakdown strength at some build orientations is relatively low and requires further optimization.

This behavior indicates that the AC breakdown process cannot be interpreted solely in terms of long-range charge transport. When the applied electric field is perpendicular to the built layers, multiple interlayer interfaces are sequentially arranged along the electric-field direction and can be approximated as a series of interfacial barriers. Charge carriers must cross these interfaces sequentially, which strongly restricts their transport and results in a relatively high AC breakdown strength. When the build layers are inclined with respect to the applied electric field, the field can be resolved into a normal component perpendicular to the layers and a tangential component parallel to them. At printing angles of 30–75°, the normal electric field component may enhance local electric field distortion around interlayer defects, while the tangential component facilitates charge migration along the relatively weak secondary sintered layers or interlayer regions. The synergistic effect of these two components promotes the formation of local breakdown paths, resulting in a lower AC breakdown strength. At 90°, the normal electric-field component across the interlayer interfaces is substantially reduced, thereby weakening the local-field distortion associated with interlayer defects and leading to a partial recovery of the AC breakdown strength. Notably, the Weibull scale parameter for AC breakdown is numerically higher at 15° than at 0°, indicating that the orientation dependence is not strictly monotonic. The 95% confidence intervals of these estimates overlap, and the present results do not establish a specific cause for this difference. Variations in pore distribution and interlayer features may affect AC breakdown, as discussed in Section 3.6 and Section 4; however, their contributions to the difference between 0° and 15° cannot be determined from the available evidence.

### 3.4. Tensile Properties

**Figure 7:**
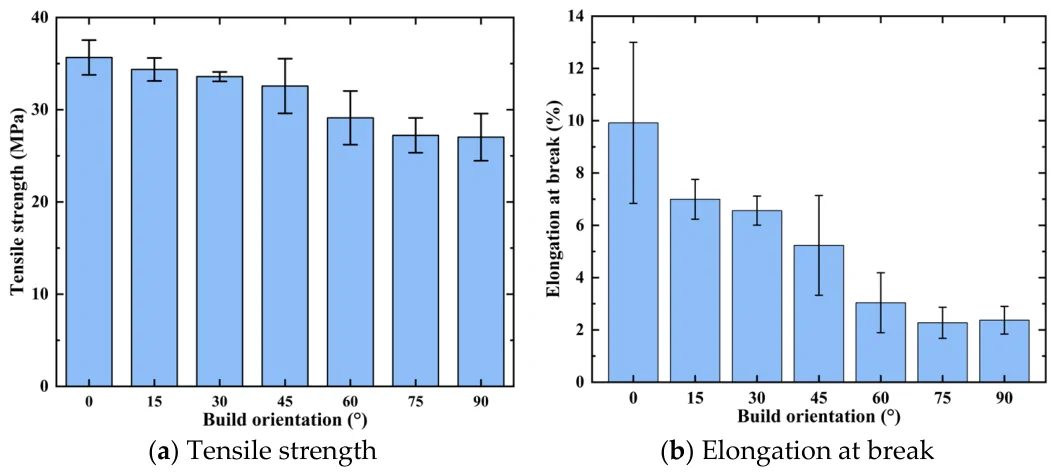

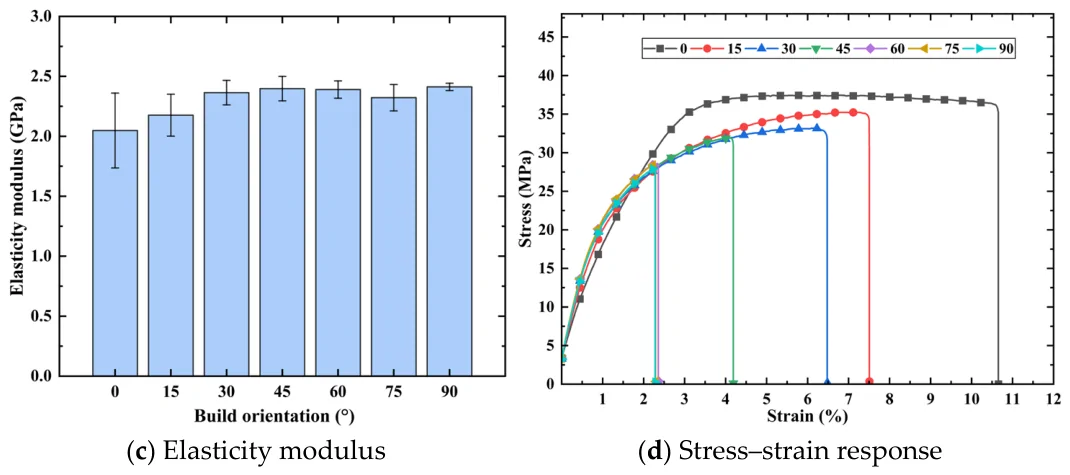
Tensile test results of specimens with different build orientations.

The tensile test results are shown in Figure 7. The tensile strength decreases as the printing angle increases, with the 0° specimen exhibiting the highest tensile strength and the 90° specimen the lowest. The tensile strength of the 0° specimen is approximately 1.3 times that of the 90° specimen. The elongation at break also generally decreases as the printing angle increases. The 0° specimens show relatively large scatter, whereas the 75° and 90° specimens exhibit similar and relatively low elongation at break. The tensile strength ratio of approximately 1.3 between the 0° and 90° specimens is lower than those reported in some previous studies [14,28], possibly due to differences in SLS equipment and processing parameters, such as laser power and scanning speed. In contrast, the tensile modulus increases from approximately 2.05 GPa at 0° to 2.41 Gpa at 90°, although the change is not strictly monotonic. The representative stress–strain curves in Figure 7d further show that the 0° specimen sustains greater deformation before fracture, whereas specimens at higher build angles generally fracture at lower strains. Thus, build orientation affects the initial stiffness and the subsequent deformation and failure behavior differently.

**Figure 8:**
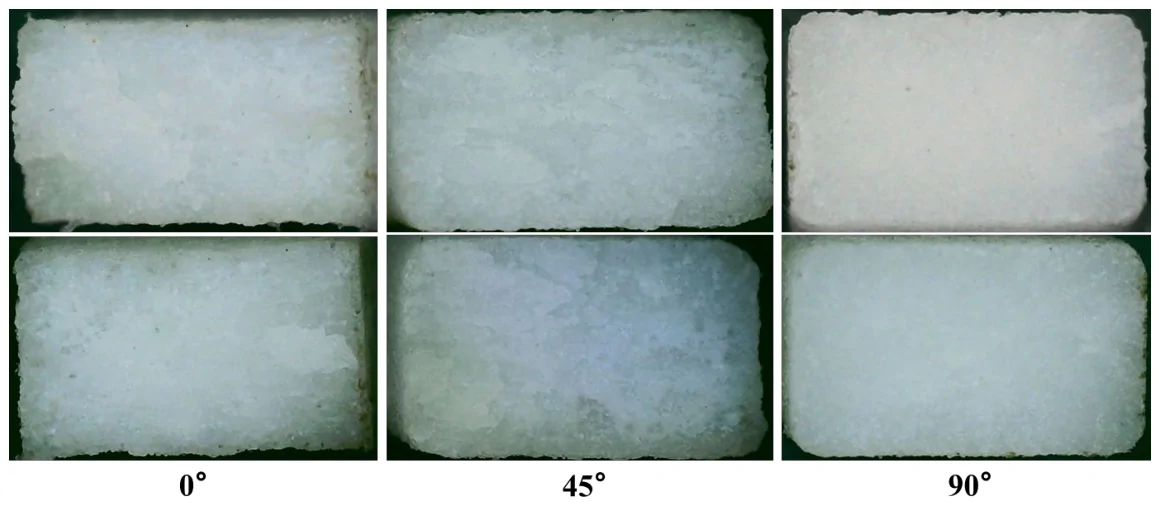
Tensile fracture surfaces of specimens with different build orientations.

The tensile fracture surfaces of representative specimens are shown in Figure 8. The 0° and 45° specimens exhibit relatively uneven and irregular fracture surfaces, whereas the 90° specimen shows a noticeably flatter surface. In particular, the fracture-surface morphology of the 90° specimen suggests a tendency toward fracture along the interlayer interfaces. These observations indicate that the relatively weak interlayer bonding formed during the SLS process contributes to the orientation-dependent tensile strength and elongation at break.

For the 0° specimen, the tensile load is approximately parallel to the build layers and is primarily carried by the relatively continuous sintered PA12 structure within each layer. Consequently, the influence of weak interlayer bonding on the overall load-bearing capacity is limited, resulting in relatively high tensile strength and elongation at break. As the printing angle increases, the tensile load develops an increasing component normal to the build layers, subjecting the relatively weak interlayer regions to greater normal tensile stress. Because these regions may contain pores and incompletely fused defects, cracks can more readily initiate and propagate along the interlayer regions, leading to gradual reductions in both tensile strength and elongation at break and more pronounced brittle fracture behavior. At a printing angle of 90°, the tensile load is approximately perpendicular to the build layers, and the load-bearing capacity is therefore predominantly governed by the interlayer bonding strength. Weak interlayer bonding, together with interlayer defects that reduce the effective load-bearing area, facilitates rapid crack propagation along the interfaces. Consequently, the 90° specimen exhibits the lowest tensile strength and a relatively low elongation at break, together with a comparatively flat interfacial fracture surface.

Although the tensile modulus of the 90° specimens is higher than that of the 0° specimens, their tensile strength and elongation at break are lower. This indicates that initial stiffness and failure behavior respond differently to build orientation. The earlier fracture of the 90° specimens is consistent with the greater influence of interlayer regions under tensile loading. The stress–strain curves show that the strain at break generally decreases as the build angle increases. At higher build angles, a larger component of the tensile load acts normal to the build layers, and interlayer bonding and associated defects may contribute to earlier fracture. At lower build angles, the load is carried to a greater extent by the continuous sintered structure within the layers, allowing the specimens to reach higher strains before fracture.

### 3.5. Thermal Properties

**Figure 9:**
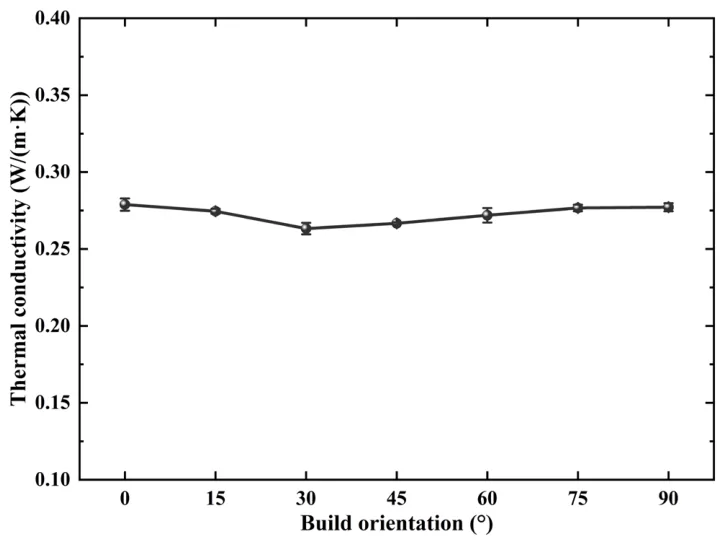
Thermal conductivity of specimens with different build orientations.

The thermal conductivity of specimens printed at different orientations is shown in Figure 9. It exhibits a non-monotonic variation, decreasing from 0.27887 W/(m·K) at 0° to a minimum of 0.26327 W/(m·K) at 30°, followed by a gradual increase to 0.27717 W/(m·K) at 90°. The maximum-to-minimum ratio is approximately 1.06, and the difference between the 0° and 90° specimens is only 0.61%. The coefficients of variation range from 0.28% to 1.74%, indicating low scatter within each orientation. These results indicate a relatively weak dependence of effective thermal conductivity on build orientation.

The limited variation in thermal conductivity indicates that build orientation has a relatively small effect on the thermal transport of the SLS-fabricated PA12 specimens. The minor differences among orientations may be associated with variations in porosity and interlayer fusion quality, which affect the continuity of heat-transfer paths and local thermal resistance.

### 3.6. Microstructure and Crystallinity of SLS-Printed PA12

**Figure 10:**
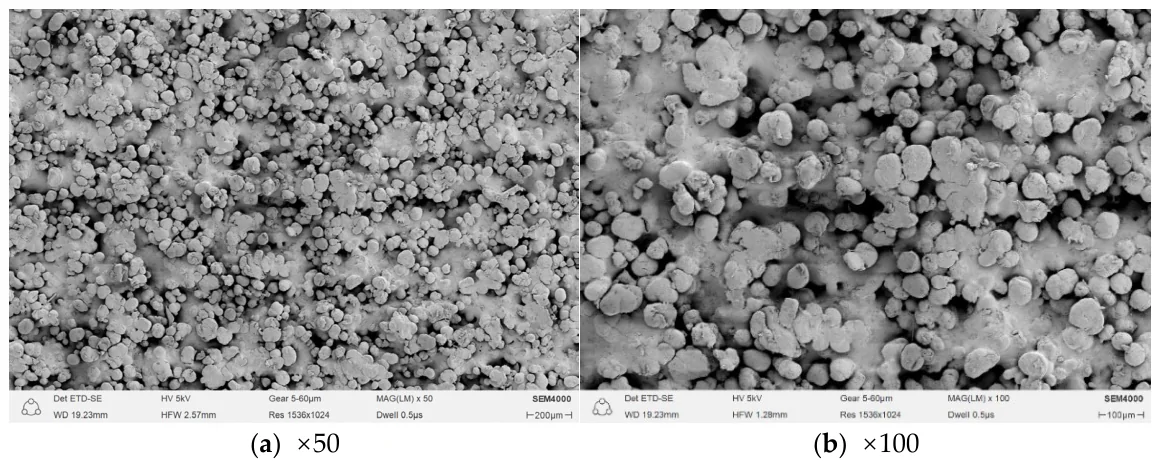
SEM images of SLS-printed PA12 specimens.

As described above, SLS-printed PA12 consists of primary and secondary sintered regions, while pores, incompletely fused regions, and weakly bonded interfaces may exist between adjacent build layers. To further characterize the microstructure, the specimens were sectioned, sputter-coated with gold, and examined using a field emission scanning electron microscope (SEM) (SEM4000, CIQTEK, Hefei, China) at an accelerating voltage of 5 kV. The results are shown in Figure 10. The SEM images were obtained from cross-sectional surfaces along the build direction to reveal the interlayer structure.

At low magnification, numerous powder particles with clearly distinguishable boundaries remain visible, indicating that some PA12 particles were not fully melted during the SLS process. Although partial melting and bonding occurred between adjacent particles, irregular interparticle pores remained. At higher magnification, sintering necks and locally fused regions can be clearly identified, together with partially melted particles and incompletely fused defects. This nonuniform particle fusion produces a heterogeneous microstructure comprising relatively dense sintered regions and porous regions, thereby weakening interlayer bonding and affecting both heat transfer and charge-carrier transport.

**Figure 11:**
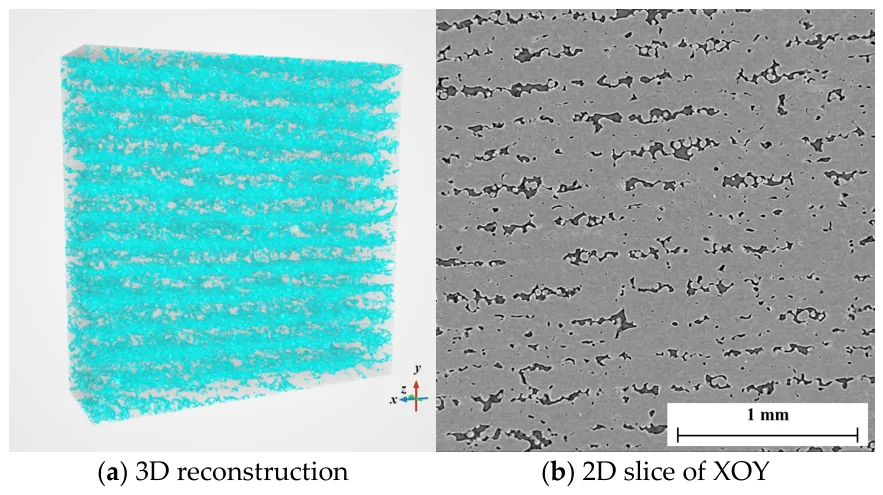
XRM results of SLS-printed PA12 specimens.

To further examine the spatial distribution of these heterogeneous regions, a 1 mm thick specimen fabricated at a build orientation of 90° was characterized by X-ray microscopy (XRM) (Zeiss Xradia 515 Versa, Oberkochen, Germany), as shown in Figure 11. The 3D reconstruction reveals numerous internal pores within the SLS-printed PA12. These defects are not completely randomly distributed but show a clear tendency to concentrate between adjacent build layers. Figure 11b presents an X-Y slice taken at the midpoint of the reconstructed volume along the *Z*-axis, corresponding to the front view in Figure 11a. The 2D slices further confirm that a large proportion of the pores are located in the interlayer regions. These XRM observations are consistent with the porous heterogeneous structure revealed by SEM and provide a microstructural basis for the anisotropic electrical, thermal, and mechanical properties of SLS-printed PA12.

**Figure 12:**
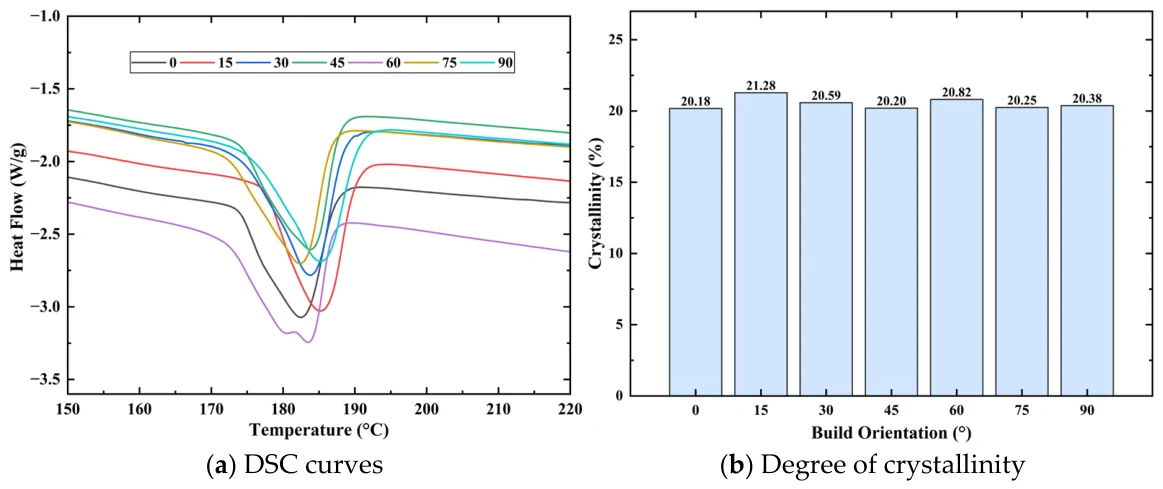
(**a**) DSC curves and (**b**) calculated degree of crystallinity of PA12 specimens fabricated at different build orientations.

To assess whether differences in crystallinity contribute to the observed anisotropy, PA12 specimens printed at seven build orientations were analyzed using a Mettler-Toledo DSC 3 instrument (Greifensee, Switzerland). Samples weighing approximately 2~5 mg were placed in 40 μL aluminum crucibles and heated from 0 to 250 °C at 10 K/min under nitrogen flowing at 50 mL/min. Crystallinity was calculated from the melting enthalpy using 209.3 J/g as the reference value for fully crystalline PA12. As shown in Figure 12, all specimens exhibit a single melting peak at approximately 181~184 °C, consistent with the melting point reported in Table 1. Crystallinity ranges from 20.18% to 21.28%, with an average of (20.5 ± 0.4)%, and shows no systematic dependence on build orientation. These results suggest that differences in crystalline fraction are unlikely to be the primary cause of the observed electrical, thermal, and mechanical anisotropy.

The XRM observations provide qualitative information on the internal pore distribution. Pore volume fraction, mean pore size, and orientation distribution have not been quantified in this study. These descriptors are needed to establish quantitative relationships between pore structure and macroscopic properties, and their determination will be addressed in future work.

## 4. Mechanism Analysis

**Figure 13:**
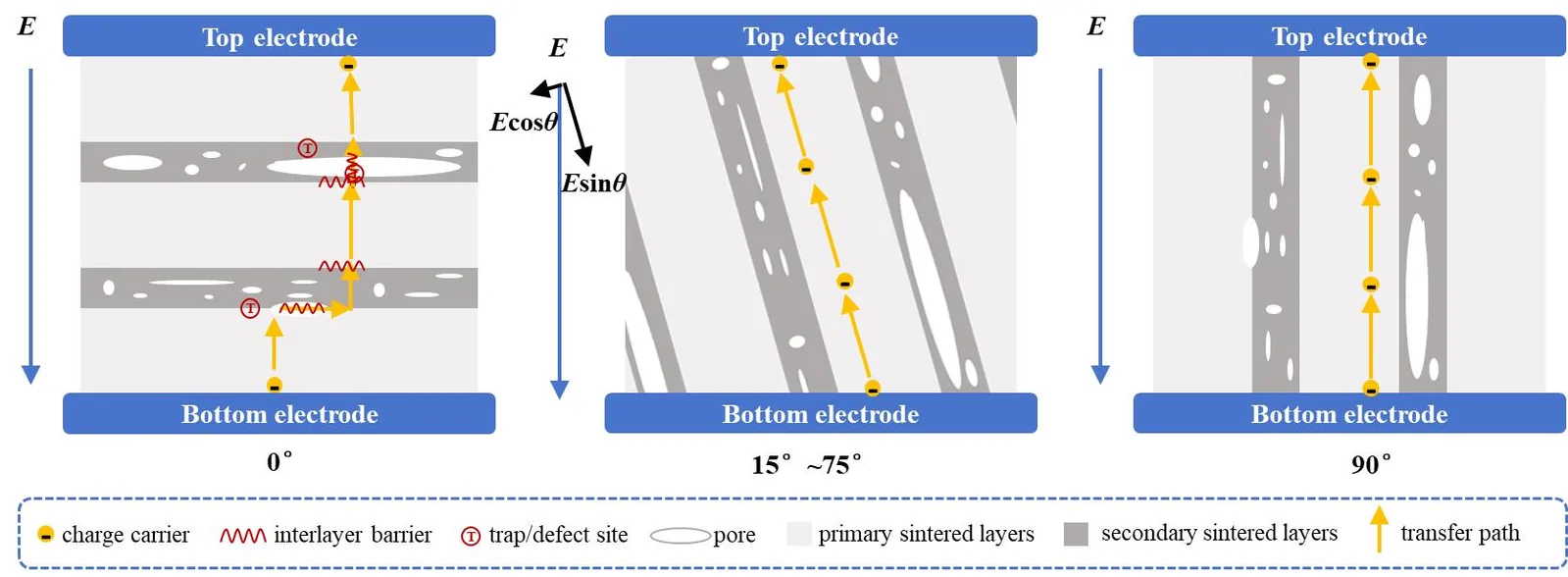
Schematic illustration of the proposed qualitative mechanism for the orientation-dependent properties of anisotropy in SLS-printed PA12.

Based on the microstructural observations and the electrical, thermal, and mechanical results described above, a qualitative interpretation of the anisotropic behavior of SLS-printed PA12 is proposed, as illustrated in Figure 13. The schematic is intended to explain the experimentally observed orientation-dependent trends rather than to serve as a quantitative predictive model.

When the electric field is perpendicular to the build layers (0°), charge carriers must successively cross multiple interlayer interfaces. Interlayer pores, incompletely sintered regions, and interfacial traps hinder charge transport, resulting in relatively high volume resistivity and DC breakdown strength. Meanwhile, the heterogeneous layers are approximately arranged in series along the direction of the electric field, resulting in a relatively low effective dielectric constant. For AC breakdown, the successive interlayer interfaces also hinder the development of a continuous breakdown path, resulting in a relatively high AC breakdown strength at 0°.

When the build layers are inclined with respect to the electric field (15–75°), the applied field can be resolved into a component parallel to the build layers, Esinθ, and a component normal to the layers, Ecosθ. The parallel component provides a more favorable pathway for charge transport along the sintered or interlayer regions, resulting in an overall decrease in volume resistivity and DC breakdown strength as the printing angle increases. Meanwhile, as the dielectric structure gradually changes from a series-dominated to a more parallel configuration, the effective dielectric constant increases. For AC breakdown, the combined effects of local-field distortion induced by the normal component and charge transport promoted by the parallel component lead to lower breakdown strengths at intermediate printing angles.

When the electric field is parallel to the build layers (90°), charge carriers can migrate more readily along the relatively continuous sintered regions with minimal crossing of interlayer interfaces. Consequently, the volume resistivity and DC breakdown strength reach their lowest values. In this configuration, the heterogeneous dielectric regions are more nearly arranged in parallel, resulting in a higher effective dielectric constant. Meanwhile, the electric field component normal to the interlayer interfaces is substantially reduced, thereby suppressing defect-induced local-field distortion and producing a partial recovery of the AC breakdown strength relative to the intermediate printing angles.

The PA12 specimens exhibit low thermal conductivity across all build orientations, with a maximum-to-minimum ratio of approximately 1.06. This indicates that build orientation has a relatively small effect on thermal conductivity under the present experimental conditions. The minor differences among orientations may be associated with variations in pore distribution and interlayer fusion quality. In terms of mechanical properties, interlayer pores and weakly bonded regions reduce both the effective load-bearing area and the interlayer bonding strength. As the printing angle increases, the component of tensile stress normal to the build layers increases, facilitating crack initiation and propagation along weak interlayer regions, thereby reducing both tensile strength and elongation at break.

The present mechanistic interpretation is based primarily on microstructural observations and macroscopic property variations and therefore remains qualitative. The complex spatial distributions of pores and interlayer interfaces formed during SLS may not be adequately represented by effective-medium approaches, such as series–parallel, Maxwell–Garnett, or Bruggeman models. However, the available observations provide insufficient quantitative information on three-dimensional pore morphology, connectivity, and interlayer fusion quality to determine model parameters reliably. Moreover, this study focused on build orientation without systematically varying other processing parameters, limiting the establishment of quantitative processing–microstructure–property relationships. Further work should quantify these structural features and their dependence on processing conditions, then incorporate them into effective-property models to predict material properties at different build orientations.

## 5. Conclusions

This study systematically investigated the effects of build orientation on the electrical, thermal, and mechanical properties of SLS-printed PA12 and interpreted the observed anisotropy through microstructural characterization and mechanism analysis. The results demonstrate that SLS-printed PA12 exhibits pronounced orientation-dependent properties, providing a theoretical basis for its structural design and performance optimization in high-voltage insulation applications. The main conclusions are as follows:

(1) SLS-printed PA12 shows pronounced electrical anisotropy. With increasing printing angle, the DC breakdown strength and volume resistivity generally decrease, whereas the relative permittivity gradually increases. The AC breakdown strength and dielectric loss tangent are also affected by printing orientation, although their variations are not strictly monotonic.
(2) The thermal conductivity varies only slightly among specimens with different build orientations but generally increases with increasing printing angle, while also being affected by the distribution of internal pores and other defects. In contrast, the tensile strength gradually decreases as the printing angle increases, indicating clear mechanical anisotropy.
(3) Microstructural characterization reveals numerous interlayer pores and incompletely sintered regions within the SLS-printed PA12, exhibiting a distinct layered distribution. These heterogeneous interlayer structures modify the pathways of charge, heat, and load transport and are the primary structural origin of the anisotropic electrical, thermal, and mechanical properties.

Although the present results provide a material-level basis for selecting the build orientation of SLS-printed PA12 insulating components, the experiments were limited to laboratory-scale specimens under controlled conditions. Future work will evaluate the effects of humidity, temperature, sustained electrical stress, and long-term aging on the electrical, thermal, and mechanical properties of the printed material. To meet the requirements of high-voltage applications, the material and printing process need to be optimized to improve the breakdown strength. In addition, future work will quantitatively investigate the effects of multiple processing parameters on pore structure and interlayer fusion and develop models linking these microstructural features to the macroscopic properties of SLS-fabricated materials.

## Figures and Tables

**Table 1:** Properties of the PA12 Material.

Properties	Value
Density (g/cm^2^)	0.98
Tensile strength (Mpa)	46
Tensile modulus (Mpa)	1600
Elongation at break (%)	36
Melting point (℃)	184
HDT at 1.8 Mpa (℃)	52

**Table 2:** SLS processing parameters.

Processing Parameter	Value
Laser power (W)	75
Scanning speed (mm/s)	12,700
Scan spacing (mm)	0.3
Layer thickness (mm)	0.12
Laser spot radius (mm)	0.25

**Table 3:** Weibull parameters and 95% confidence intervals for the DC breakdown strengths.

Build Orientation (°)	α (kV/mm) [95% CI]	β [95% CI]
0	24.39 (23.25~25.58)	13.74 (8.62~21.90)
15	13.48 (13.14~13.83)	25.44 (15.12~42.82)
30	13.31 (12.77~13.88)	15.54 (9.22~26.20)
45	12.36 (11.41~13.39)	8.20 (5.10~13.18)
60	10.10 (9.78~10.43)	20.28 (12.32~33.38)
75	9.88 (9.31~10.49)	11.04 (7.13~17.10)
90	8.96 (8.72~9.21)	24.18 (15.02~38.91)

**Table 4:** Weibull parameters and 95% confidence intervals for the AC breakdown strengths.

Build Orientation (°)	α (kV/mm) [95% CI]	β [95% CI]
0	10.63 (9.85~11.47)	8.63 (5.36~13.88)
15	12.26 (11.23~13.39)	7.44 (4.55~12.15)
30	6.32 (6.07~6.58)	16.25 (9.81~26.92)
45	6.46 (6.15~6.78)	13.52 (8.66~21.11)
60	6.31 (6.11~6.51)	20.35 (12.07~34.32)
75	5.67 (5.34~6.02)	11.19 (7.42~16.89)
90	8.03 (7.63~8.46)	12.80 (7.98~20.54)

## Data Availability

The original contributions presented in this study are included in the article. Further inquiries can be directed to the corresponding author.

## Abbreviations

The following abbreviations are used in this manuscript:

SLS	Selective laser sintering
PA12	Polyamide 12
3D	Three-dimensional
SLA	Stereolithography
FDM	Fused deposition modeling
SEM	Scanning electron microscope
XRM	X-ray microscopy
